# Osteogenic differentiation of 3D-printed porous tantalum with nano-topographic modification for repairing craniofacial bone defects

**DOI:** 10.3389/fbioe.2023.1258030

**Published:** 2023-08-21

**Authors:** Chuxi Zhang, Zhongwei Zhou, Nian Liu, Jiangping Chen, Jinyang Wu, Yong Zhang, Kaili Lin, Shilei Zhang

**Affiliations:** ^1^ Shanghai Key Laboratory of Stomatology and Shanghai Research Institute of Stomatology, Department of Oral and Cranio-Maxillofacial Surgery, National Clinical Research Center, Shanghai Ninth People’s Hospital, College of Stomatology, Shanghai Jiao Tong University School of Medicine, Shanghai, China; ^2^ Ningxia Key Laboratory of Oral Diseases Research, Department of Oral and Maxillofacial Surgery, General Hospital of Ningxia Medical University, Institute of Medical Sciences, Yinchuan, Ningxia, China

**Keywords:** oral and cranio-maxillofacial, tantalum, three-dimensional printing, alkali-heat-treatment, nano-topography, osteogenesis

## Abstract

**Introduction:** Congenital or acquired bone defects in the oral and cranio-maxillofacial (OCMF) regions can seriously affect the normal function and facial appearance of patients, and cause great harm to their physical and mental health. To achieve good bone defect repair results, the prosthesis requires good osteogenic ability, appropriate porosity, and precise three-dimensional shape. Tantalum (Ta) has better mechanical properties, osteogenic ability, and microstructure compared to Ti6Al4V, and has become a potential alternative material for bone repair. The bones in the OCMF region have unique shapes, and 3D printing technology is the preferred method for manufacturing personalized prosthesis with complex shapes and structures. The surface characteristics of materials, such as surface morphology, can affect the biological behavior of cells. Among them, nano-topographic surface modification can endow materials with unique surface properties such as wettability and large surface area, enhancing the adhesion of osteoblasts and thereby enhancing their osteogenic ability.

**Methods:** This study used 3D-printed porous tantalum scaffolds, and constructed nano-topographic surface through hydrothermal treatment. Its osteogenic ability was verified through a series of *in vitro* and *in vivo* experiments.

**Results:** The porous tantalum modified by nano-topographic surface can promote the proliferation and osteogenic differentiation of BMSCs, and accelerate the formation of new bone in the Angle of the mandible bone defect of rabbits.

**Discussion:** It can be seen that 3D-printed nano-topographic surface modified porous tantalum has broad application prospects in the repair of OCMF bone defects.

## 1 Introduction

Oral and cranio-maxillofacial surgery (OCMS) is a surgical specialty that concentrates on the treatment of lesions in the oral, maxillofacial, facial, or head and neck areas (Bell, 2010) with complex anatomy, important physiological functions, and aesthetic requirements (Kim J.-W. et al., 2016; Rachmiel et al., 2017). In fact, congenital or acquired defects and malformations in the craniomaxillofacial (CMF) region (Rudman et al., 2011; Seok et al., 2016; Oh, 2018), especially bone defects, stemmed from traffic accidents, sports injuries, congenital malformations, tumor resection, surgical accidents and periodontal diseases not only severely affect the normal function and facial appearance, but also brings harm to the physical and mental health of patients.

However, the reconstruction of CMF bone defects is extremely challenging in the global medical system due to its stringent requirements in shape and high risk of postoperative infection in the selection of repairing materials. An ideal material needs to satisfy several conditions. Firstly, the restoration with high biocompatibility is required to prevent from any adverse reactions such as immune rejection in the human body, and the material is supposed to provide mechanical support for the defect area with excellent mechanical performance. Appropriate surface properties and porosity are also needed to simulate the composition, structure and performance of biological bone tissue, while the specific pore size and porosity are conducive to the generation of fresh and orderly vascular systems throughout the defect spaces, facilitating the transfer of nutrients and cells to the newborn bones. In addition, the induction and conduction of bones are instrumental in inducing the mesenchymal cells adjacent tissue to differentiate into osteoblasts or facilitating the crawling of nearby bone tissue (Gundu et al., 2022).

Among all bone defect implant materials used in clinical, metal materials have strong plasticity and can be adjusted appropriately during surgery to achieve better surgical results, and Ti6Al4V is currently the most widely used metal implant material. Huang et al. (2022) demonstrated through cell experiments that tantalum scaffolds with the same structure have better osteogenic ability than Ti6Al4V scaffolds. Fan et al. (2021) tested the biomechanical properties of tantalum and titanium scaffolds in reconstructing bone defects, proving that tantalum scaffolds outperform titanium scaffolds in terms of compression and deformation, and have biomechanical properties closer to bone scaffolds. In addition, the research of Schildhauer et al. (2006) proved that tantalum scaffold has lower *Staphylococcus aureus* adhesion than titanium alloy. The above studies have shown that tantalum has better mechanical properties, osteogenic ability, and microstructure compared to titanium alloys, making it a potential substitute material for bone repair. Tantalum has been widely applied in orthopedics such as hip, knee arthroplasty and spine surgery as a new type of biomedical implant material, which is stable within the pH and voltage ranges *in vivo*. However, tantalum is generally biologically inert in the human body and encounters limitations in inducing the formation of new bones indicating that directly integration with the surrounding bone is impractical. Clinically, it will take 3–6 months to ensure the sufficient osseointegration if necessary. Therefore, how to promote osseointegration and shorten the time has been heated debated for tantalum.

The surface characteristics of materials, such as surface morphology and surface chemistry are strongly relevant to the biological behavior of cells and deeply influenced the performance (Ponche et al., 2010). It has been reported (Vitte et al., 2004) that the long-term adhesion of cells is associated with the surface morphology of materials, especially the roughness, while short-term adhesion is mainly controlled by surface chemistry.

Most cells in the body (except blood cells) adhere to the extracellular matrix (ECM), while the bone-forming and degrading cells, i.e., osteoblasts and osteoclasts in the bone tissue attach to and grow on the ECM, which consists of proteins and calcium phosphate-rich mineral proteoglycans. Only when cells adhere normally can they continue to grow and differentiate (Anselme et al., 2010). The first interaction between the cell and the material surface will determine the binding quality of the tissue-implant interface, and the surface will be covered with water and proteins after a few seconds of contact between the material and the body fluid by which the cells sense the surface characteristics of the ECM. Cells adhere with the help of physicochemical interactions such as ionic force and van der Waals force firstly, and then by various biomolecules. The most critical part in this process is the surface receptor also called integrin, which is a transmembrane molecule interacting with the ECM outside the cell and the cytoskeleton molecules and adhesion sites inside in order to transfer the information between the both sides. Therefore, signals are transmitted from the ECM to the nucleus through the biochemical signal transduction pathways for the sake of protein aggregation and phosphorylation (Siebers et al., 2005). It seems reasonable to regulate the differentiation of stem cells by modifying the extracellular environment.

Nano-technology is widely used in surface modification of implants (Manivasagam et al., 2022). Due to the unique surface properties of nanomaterials such as wettability, large surface area, proteins will attach to the nano-surface and form a protein layer, acting as a biological medium for the interaction between the nanomaterials and cells (Zhang et al., 2020). The unique surface properties of nano biomaterials increase protein adhesion. The enhanced protein adsorption layer continues to increase the cell adhesion through signal recognition triggered by integrin and ultimately affect subsequent cell reactions, such as cell migration and osteoblastic differentiation (Huang et al., 2016). After the nanomaterials are overlapped by albumin, laminin, or collagen, the adhesion of osteoblasts to the nanomaterials is significantly enhanced. In addition, the adsorption kinetics of proteins vary with different chemical and physical properties. Specific types of proteins can better adhere to the surface of the nanomaterials, selectively mediating the adhesion of osteoblasts (Webster et al., 2000).

Researchers have developed many methods to prepare the nano-topographic surface, such as thin film deposition, physical and chemical vapor deposition, chemical etching, nanoimprinting, photolithography, electron beam or nanosphere lithography, etc. (Babuska et al., 2022). Among these methods, alkali heat treatment enables to significantly improve in the hydrophilicity of the material surface (Kim H. S. et al., 2016), which facilitates the interaction of cells with the implant surface. Moreover, alkali heat-treated metallic tantalum effectively induces extracellular bone matrix mineralization by rapid hydroxyapatite deposition in simulated body fluids (Miyazaki et al., 2000).

Finished biomaterials processed by slicing are not adequate for the anatomical structure of bone defects and the demands of defects with critical sizes and maxillofacial aesthetics, and they are mostly used for skull repair (Jardini et al., 2014). The development of 3D printing technology has largely solved these challenges, thus providing a new direction for the treatment of maxillofacial bone defects (Jones, 2012).

**FIGURE 1:**
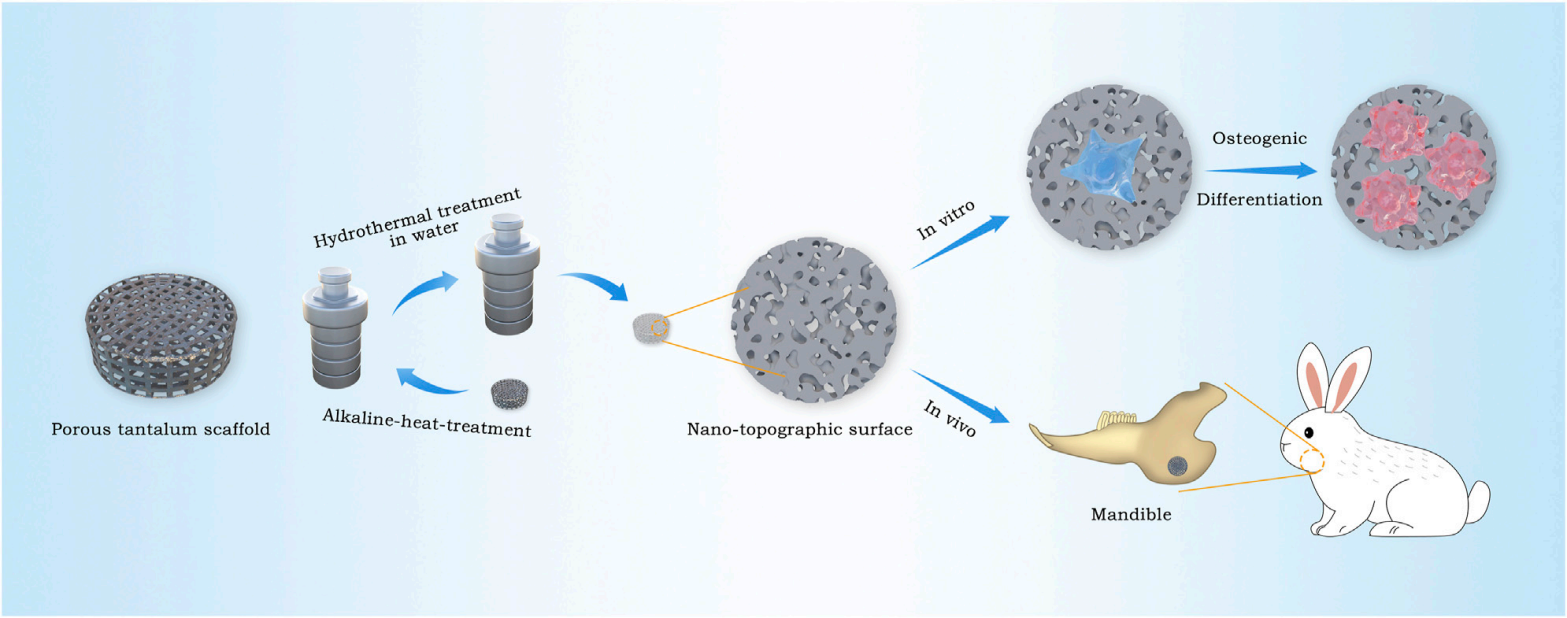
Schematic illustration for the construction procedure of nano-topographic surface tantalum scaffold and its application of osteogenic effects *in vitro* and vivo.

In this work, 3D printing technology is used to produce porous tantalum scaffolds, then nano-morphology on the surface of the scaffolds is constructed by alkali heat treatment with NaOH solution and the following hydrothermal treatment in deionized water in order to realize the rapid bone integration, high compatibility with the elastic modulus of human bones, and satisfy personalized demands. The surface characteristics of 3D-printed porous tantalum scaffolds modified in nano-topographic are systematically analyzed. Further research about the biological reaction *in vitro* and the ability of bone integration *in vivo* of nano-topographic modified 3D-printed porous tantalum scaffold were studied by co culturing rat bone marrow stem cells (BMSCs) *in vitro* and repairing the bone defect of the rabbit mandible angle *in vivo* (Figure 1).

## 2 Materials and methods

### 2.1 Materials

The samples are prepared by Ningxia Dongfang Zhizao Technology Co., Ltd., and the preparation method is as follows. Firstly, the scaffold model is constructed by Geomagic Studio 2014 (Geomagic Inc., Morrisville, NC, United States), and 3D printed by Huashu FS271M laser printer (Huanan Farsoon High Technology Co., Ltd., China) using Tantalum medical powder with an average particle size of 15–53 μm. Every layer of the porous scaffolds was processed by laser selective melting.

The scaffold with a diameter of 10 mm, a thickness of 2 mm is used for *in vitro* cell experiments, while the one with a diameter of 6 mm, a thickness of 2 mm is prepared for *in vivo* animal experiments. The pore size of these two types is 400–500 μm, and the porosity is 70%–80% with <20 GPa elastic modulus. All samples were ultrasonically cleaned in acetone, anhydrous ethanol and distilled water for 15 min.

To construct nano-topographic surface, the 3D-printed porous tantalum scaffolds were immersed in a 5 mmol/L NaOH solution and subjected to hydrothermal treatment at 80°C for 6 h in a Teflon-lined autoclave. After thoroughly cleaned with deionized water using ultrasonic cleaner, the samples were wet oxidized at 200°C for 4 h in deionized water (Wang et al., 2018). The samples obtained before and after the hydrothermal treatment were labeled as Ta-3D and Ta-nano respectively, where the 3D samples were used as controls. Autoclaving steam sterilization (0.21 MPa, 134°C) was performed prior to *in vitro* and *in vivo* studies.

### 2.2 Methods

#### 2.2.1 Surface characterization of the samples

The surface morphology of the prepared samples was observed by a scanning electron microscope (SEM, Hitachi SU8220, Japan). The chemical phases on the surface of two groups of materials were identified by X-ray diffractometry (XRD, Buker D8 advance, Germany) using a Cu Kαx-ray source with a 2θ scan range of 20°–60° and a scan rate of 2°/min. The compositions of the samples were analyzed by energy dispersive x-ray spectrometry (EDS, EBSD X-MAXN, OXFORD, Britain). The surface wettability of the material is measured by a contact angle meter (SL200KS, SOLON TECH, China).

#### 2.2.2 *In vitro* experiments

##### 2.2.2.1 Rat BMSCs separation and culture

Male rats at 3 weeks of age were selected to extract BMSCs cells. The rats were cervically dislocated and placed in 70% ethanol for immersion. The executed rats were placed supine on a dissecting board, a skin augmentation was made on the abdomen with forceps, a small incision about 1 cm long was made with sterile dissecting scissors, and the skin of the limbs and feet were peeled from the wound to expose the lower abdomen and legs. The foot was removed by making an incision from below the ankle joint, and an incision was made along the iliac bone at the hip bone to separate the femoral head from the hip bone, and an incision was made in the middle of the hip bone to separate the two iliac bones. Using vascular forceps and small scissors, the muscles of the femur, tibia and ilium were carefully removed. After separating all bones, they are placed in Phosphate buffer solution (PBS) and moved to the ultra-clean bench for the remaining separation steps. Prepare Petri dishes containing 10 mL of prepared α-MEM medium (Shanghai BasalMedia Technologies Co., Ltd., Shanghai, China) (containing 10% fetal bovine serum (FBS, Gibco, United States), 1% penicillin and streptomycin double antibodies). Make small incisions of approximately 1–2 mm both proximal and distal to the bone, aspirate 3 mL of the configured α-MEM medium with a 5 mL syringe, rinse the bone, flush the rinse solution into the Petri dish, and wash the bone cavity thoroughly with the syringe at least three times until the bone appears white, indicating that all bone marrow has been flushed. The medium was incubated in an incubator at 37°C and 5% CO_2_. Primary BMSCs cells were cultured for 72 h. Fresh medium was added to remove non-adherent cells, and the remaining adherent cells were mainly bone marrow mesenchymal stem cells. After that, the medium was changed every 2 days until the cell fusion reached 80% and the cells were ready for passaging. BMSCs cells of three to five generations were selected for the following experiments.

##### 2.2.2.2 Cell adhesion observation

In a 48-well plate, BMSCs cells were inoculated onto each sample at a density of 2×10^4^/well. After 24 h of incubation, the cells were rinsed twice with PBS, then fixed for 24 h at 4°C by adding 4% paraformaldehyde, and rinsed three times with PBS. The cells were then dried in a vacuum freeze dryer (CoolSafePro110-4, LABOGENE, Denmark) for 24 h. After gold spraying, the morphology and adhesion of cells on the surface of porous metallic tantalum material were observed by scanning electron microscopy at 3.0 kV.

##### 2.2.2.3 Cell live/dead assay

The toxicity of different samples to cells was studied by live/dead staining. Ta-3D and Ta-nano porous materials were spread on 48-well plates, where 4 wells of each material. BMSCs were seeded on the materials at a density of 1×10^4^ cells/well, and they were cultured for 3 days. Staining was performed with Calcein/PI cell activity and cytotoxicity assay kit (Shanghai Maokang Biotechnology Co., Ltd., Shanghai, China) and the observation and counting are carried out under fluorescent microscope.

##### 2.2.2.4 Cell proliferation

BMSCs were inoculated at a cell density of 5,000 cells/well on each group of material, and 0.5 mL of α-MEM complete medium was added to each well. And they were incubated for 1/4/7 days. At each time point, the medium was removed and, and the samples were washed 2–3 times with PBS and transferred to a new 48-well plate with sterilized forceps, and 0.5 mL of α-MEM complete medium containing 10% CCK8(Cell Counting Kit-8, Dojindo Laboratories, Japen) reagent was added to each well. Away from light, the plate was incubated for 2 h in the incubator, and then transferred to a 96-well plate with 100 μL per well. The absorbance OD value was determined at 450 nm in an enzyme marker.

##### 2.2.2.5 The alkaline phosphate (ALP) activity assay

Ta-3D and Ta-nano porous materials were spread in two 48-well plates with 4 wells per material, and BMSCs were seeded on the material at a density of 2×10^4^ cells/well. After 4 and 7 days of incubation, the material was rinsed 2–3 times with PBS, transferred to a new 48-well plate. After covering the materials with 0.5–1 mL per well 1% TritonX-100 (Beyotime, China) for 30–40 min, the lysate was then transferred to a 15 mL centrifuge tube using a micropipette and centrifuged at 12,000 rpm/min for 10 min at 4°C to obtain the supernatant. ALP activity was determined using the ALP kit (JianCheng Bioengineering Institute, Nanjing, China) and total protein concentration was determined using the BCA protein assay kit (Beyotime, China), and all operations were performed on ice. The AKP standard curve was used for calculation of the assay results.

##### 2.2.2.6 Alkaline phosphatase staining assay

Ta-3D and Ta-nano porous scaffolds were spread in 48-well plates, 4 wells of each material, for a total of 2 plates. BMSCs were seeded on the materials with a density of 2×10^4^ cells/well. Due to the dark color of the scaffolds, alkaline phosphatase activity staining purple was difficult to develop, so the cells were cultured for 4 and 7 days, and then after complete digestion of the cells from the scaffolds using 0.25% trypsin digestion solution (Gibco, United States), they were transferred to new 48-well plates and cultured for another 24 h. The intracellular ALP was characterized using the BCIP/NBT alkaline phosphatase color development kit (Beyotime, China). The surface was observed and photographed using a stereomicroscope.

##### 2.2.2.7 Alizarin red staining (ARS) Assay

Alizarin red staining was used to assess the extent of ECM mineralization. BMSCs were seeded on the materials at a density of 1×10^4^ cells/well. The 0.2% ascorbic acid, 1% sodium β-glycerophosphate and 0.01% dexamethasone were added to complete α-MEM medium to prepare osteogenic induction medium. When the cell fusion of BMSCs reached 50%–70%, the α-MEM culture medium was aspirated and the osteogenic induction medium was added, and the medium was changed every 2 days to induce the deposition of calcium salts on the cell surface and the formation of calcium nodules. The BMSCs were cultured for 21 days on the surface of each of the two groups of samples, and then fixed with 4% paraformaldehyde for 60 min. After staining with alizarin red staining solution (2%, pH 8.3) (Beyotime, China) for 10 min, the excess dye was washed 3–5 times with phosphate buffer, and then observed and photographed under a body view microscope.

##### 2.2.2.8 Quantitative Real-Time PCR

The expression levels of osteogenic genes were analyzed using fluorescence real-time quantitative PCR to examine the expression of ALP, BMP-2, BSP and COL-1. BMSCs were seeded on the materials at a density of 2×10^4^ cells/well and cultured for 4 and 7 days. Total RNA was extracted using Trizol reagent (Invitrogen, United States). cDNA was then synthesized by Prime-Script™ RT kit (Takara Bio, Japan). The real-time PCR procedure was performed by using SYBR green PCR reaction mix (Roche, Basel, Switzerland) on the Light Cycler^®^ 96 Real-Time PCR System (Roche, Switzerland). All the above experiments were performed on ice. All expression levels of target genes were normalized to GAPDH and related genes were quantified using the 2^−ΔΔCT^ method. The primers are listed in Table 1.

**TABLE 1:** Primer sequences utilized for RT-PCR.

Gene	Primers (F = forward; R = reverse)
GAPDH	F:5′-GAAGGTGAAGGTCGGAGTC-3′
	R:5′-GAAGATGGTGATGGGATTTC-3′
ALP	F: 5′-TAT​GTC​TGG​AAC​CGC​ACT​GAA​C-3′
	R: 5′-CAC​TAG​CAA​GAA​GAA​GCC​TTT​GG-3′
BMP-2	F: 5′-GAA​GCC​AGG​TGT​CTC​CAA​GAG-3′
	R: 5′-GTG​GAT​GTC​CTT​TAC​CGT​CGT-3′
BSP	F: 5′-AGA​AAG​AGC​AGC​ACG​GTT​GAG​T-3′
	R: 5′-GAC​CCT​CGT​AGC​CTT​CAT​AGC​C-3′
COL-1	F: 5′-GCC​TCC​CAG​AAC​ATC​ACC​TA-3′
	R: 5′-GCA​GGG​ACT​TCT​TGA​GGT​TG-3′

##### 2.2.2.9 Western blot analysis

Osteogenic protein expression (COL-1) in BMSCs was assessed using Western blot. BMSCs were seeded on the materials at a density of 2×10^4^ cells/well and cultured for 7 days. Cells on the scaffold were treated with RIPA buffer containing protease and phosphatase inhibitors (Shanghai Epizyme Biomedical Technology Co., Ltd, China) on ice, and the lysate was transferred to a 1.5 mL EP tube and centrifuged at 4°C. The total protein in the supernatant denatured with protein loading buffer (Shanghai Epizyme Biomedical Technology Co., Ltd, China) was separated by SDS-PAGE, and then transferred to Polyvinylidene fluoride (PVDF) membrane (Millipore, United States). The membrane was blocked in 5% BSA dissolved in TBST at room temperature for 1 h, and then incubated overnight with primary antibodies at 4°C. After rinsing three times in TBST, the membrane was incubated at room temperature in a second antibody conjugated with fluorescence for 1 h. Protein bands detected using an infrared laser imaging system (Odyssey,United States). β-Actin serves as a reference for this study.

#### 2.2.3 *In Vivo* studies

##### 2.2.3.1 Animal Surgical Implantation

Eight healthy adult male New Zealand white rabbits were randomly divided into four groups, while the experimental group was named Ta-nano group, and the control group was called Ta-3D group. Three and 6 weeks were selected as the sample time point with four rabbits in each group at each time point. Porous tantalum prostheses were implanted in the mandibles of the rabbits, and both mandibular angles were operated on. After palpating the lower edge of the mandible and the mandibular angle, the skin and subcutaneous tissue were incised from front to back parallel to the lower edge of the mandible, approximately 3 mm above the lower edge of the mandible and at the anterior edge of the occlusal muscle, layer by layer, with an incision length of approximately 3–4 cm. According to the requirements of critical bone defect size of rabbit mandible described in the literature (Schmitz and Hollinger, 1986; Manju et al., 2018), this study chose to construct the 6 mm circular bone defect at the Angle of the mandible of rabbit. Bilateral standardized 6 mm diameter circular holes were made to create experimental defects with a stripper while drilling. Carefully embed the sterilized material into the circular hole so that the material fits closely to the bone defect area. After implantation, the wound was closed by suturing in layers. Every rabbit was reared for 2 and 4 weeks and injected with calcein and alizarin red (dose: 10 mg/kg) in the gluteus maximus muscle for fluorescent labeling, respectively. Rabbits in each group were euthanized and harvested at 3 weeks and 6 weeks after implantation.

##### 2.2.3.2 Micro-CT Analysis

The bone tissue samples were fixed in 4% paraformaldehyde for 24–48 h, and it was scanned by micro-CT (Quantum GX2 micro-CT, PerkinElmer, United States), and images and statistics were obtained using its accompanying automated bone analysis software (VAccuCT™, PerkinElmer, United States).

##### 2.2.3.3 Histological evaluation

After dehydration with an ethanol gradient and dethermalization with a series of mixtures of Technovit 7,200 resin and ethanol, samples were infiltrated in light-cured Technovit 7,200 resin, infiltrated for 3 days, and then placed in a light-cured embedding machine for 10 h. Finally, slices were cut into 300 μm size using a hard tissue slicer (E3000CP/400CS; EXACT Verteriebs, Germany) and grounded to 30–50 μm on a hard tissue grinder. Fluorescently labeled sections were observed under a laser confocal microscope (Leica, Germany) to study dynamic osteogenic information in porous implants. Van Gieson staining of the sections was performed to assess the bone growth in porous implants. Slides were rinsed with dehydrated alcohol and then observed under a optical microscope. The volume of osteogenesis within the tantalum metal porous material was calculated by ImageJ software (National Institutes of Health, United States).

#### 2.2.4 Statistics

Data were presented as mean (MD) ± standard deviation (SD). Statistical analysis was performed using *t*-test by SPSS 22.0 statistical package. The value was statistically significant only when *p* < 0.05.

## 3 Results and discussion

### 3.1 Surface characterization

**FIGURE 2:**
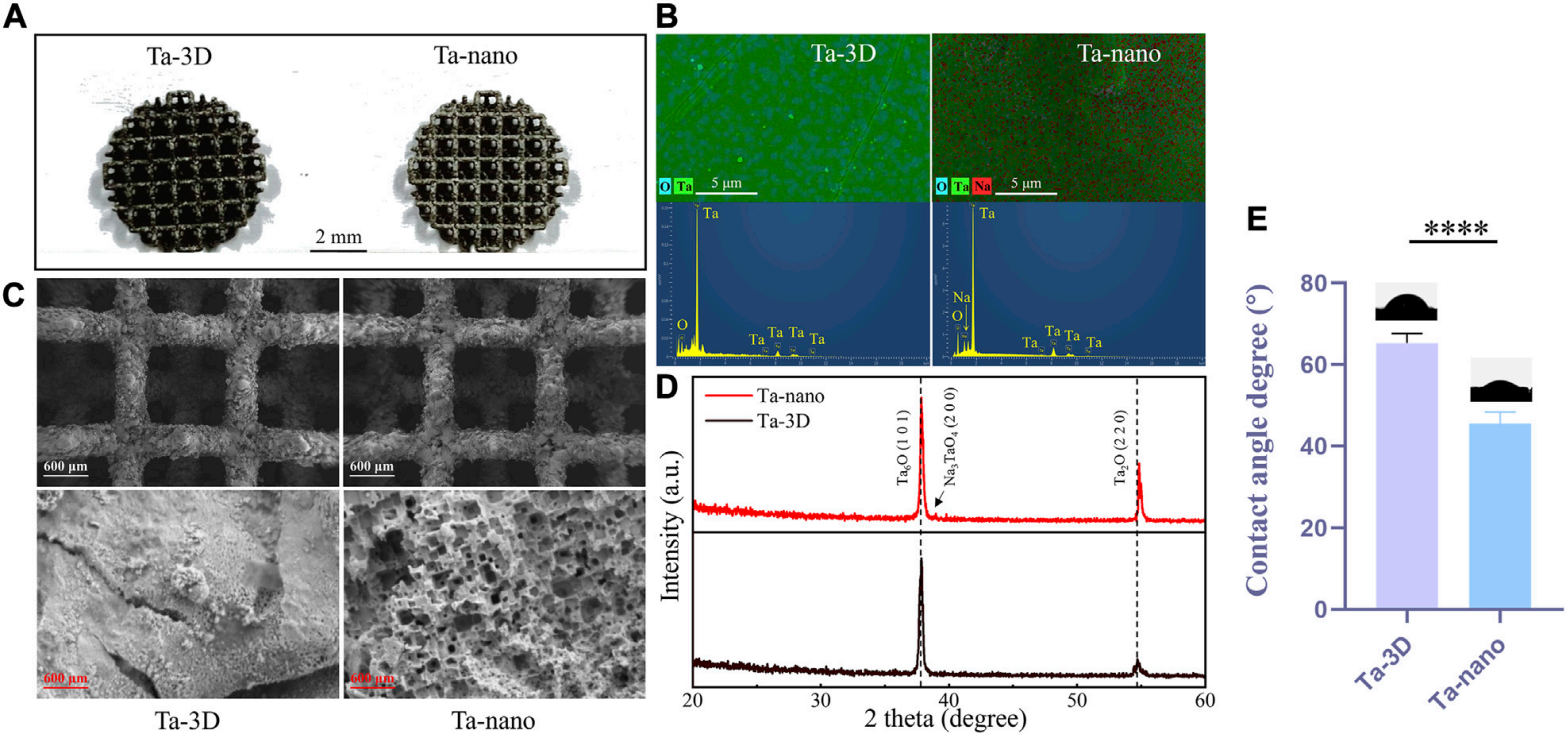
Structure and surface characterization of 3D printed porous tantalum scaffolds. **(A)** 3D-printed porous tantalum scaffolds. **(B)** SEM images of surface morphology of Ta-3D group and Ta-nano group porous tantalum scaffolds. **(C)** EDS layered image and EDS analysis of Ta-3D and Ta-nano. **(D)** XRD patterns of Ta-3D and Ta-nano. **(E)** The water contact angles of Ta-3D and Ta-nano.

Figure 2A shows three-dimensional printed porous tantalum scaffolds. The surface morphology of the porous metallic tantalum materials in the Ta-3D and Ta-nano groups was observed by SEM (Figure 2B). The Ta-3D and Ta-nano porous tantalum metal materials show the same 3D structure and similar original micron-level surface morphology at the ×30 low-magnification SEM images, which indicate that the metal powder has melted sufficiently and there is almost no Ta powder remaining on the surface of the scaffold. The porous tantalum scaffold is a regular square lattice-like structure with a side length of 1.35 mm. Under the high magnification SEM image at 30k×, the surface of the porous tantalum scaffold in the Ta-3D group is relatively flat, while the porous tantalum metal material in the Ta-nano group shows a microporous nano-topographic surface structure.

The surface chemical elemental composition of the prepared samples was further evaluated by EDS X-ray spectrometer analysis (Figure 2C), and the EDS spectra of the unprocessed tantalum metal scaffolds of Ta-3D group showed significant peaks for Ta as well as O elemental peaks. Moreover, the EDS results of the nano-surface modified tantalum metal of Ta-nano group revealed the presence of Na in addition to Ta and O.

XRD patterns of the unprocessed Ta and nanomodified porous tantalum scaffolds was shown in Figure 2D. It can be seen from the figure that the material surfaces are dominated by Ta-O oxides, which may be due to the generation of Ta_6_O/Ta_2_O oxide layer on the material surface during the laser melting. Besides, a peak corresponding to sodium tantalate (Na_3_TaO_4_) appears in the Ta-nano group due tp the reaction between Ta and the NaOH solution used in the alkali thermal operation.

The wettability of the material surface was tested using a contact angle meter. The results of the contact angle measurement are as follows (Figure 2E): the average contact angle of Ta-3D group is about 65.769°, while the average contact angle of Ta-nano group is about 42.483°. It can be seen that the nano surface modification significantly improves the wettability of tantalum metal. The wettability of biomaterials is a key characteristic that affects the initial protein adsorption and a series of subsequent cell-material interactions. Good wettability can improve the adsorption of osteoblast protein on the implant surface and increase the time of implant exposure to the cell Integrin binding domain, thereby enhancing the attachment, adhesion and further development of osteoblast precursors on the implant surface, thus accelerating bone integration (Ghezzi et al., 2021).

### 3.2 Cell adhesion

**FIGURE 3:**
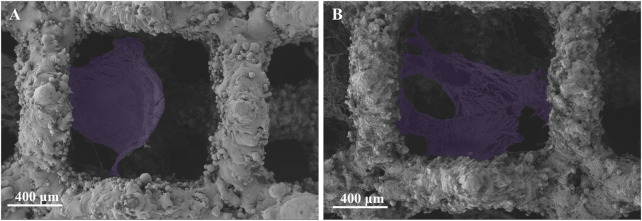
SEM images of the adherent BMSCs (purple) on Ta-3D and Ta-nano surfaces after 1 day of culture. [**(A)**: Ta-3D, **(B)** Ta-nano].

The cell morphology and adhesion of BMSCs cells cultured on porous tantalum scaffolds in the Ta-3D and Ta-nano groups for 1 day was shown in Figure 3. BMSCs cells were fully spread and adhered in both scaffold grids as seen in the 50 × SEM images. A small number of cell pseudopods were observed to adhere to the scaffold grids in the porous tantalum scaffolds of Ta-3D group. In the Ta-nano group, BMSCs cells were clearly stretched and more fully extended, with more filamentous pseudopods and lamellar footprints, indicating that the cells were more extended and adherent. As the first step in the interaction between cells and materials, cell adhesion is the foundation of cell anchoring and a prerequisite for cell diffusion and proliferation on the surface of implants (Su et al., 2022). The biological effect of surface structures on cells is determined by their sizes and it is more likely to interact between structures of the similar size; that is, micrometer-scale structures are similar in size to individual cells (tens of micrometers), while nanoscale structures formed after surface modification are almost identical to organelles, biomolecules, etc. in size, thus providing better contact guidance (Miyoshi and Adachi, 2014; Zhu et al., 2019).

### 3.3 Cell viability and proliferation

**FIGURE 4:**
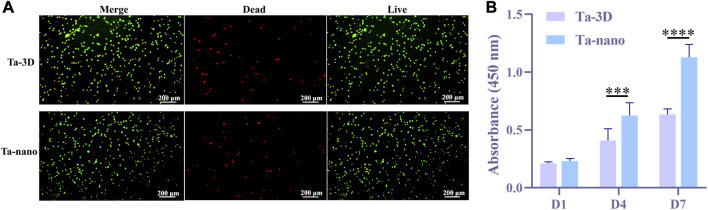
Cytotoxicity and cell proliferation. **(A)** Fluorescence images of live/dead staining after BMSCs were cultured on the surface of two samples for 3 days. **(B)** CCK-8 results showed the proliferation of BMSCs in Ta-3D and Ta-nano.P

The BMSCs cells were cultured on porous tantalum scaffolds of Ta-3D group and Ta-nano group for 3 days and then stained for live-dead staining, and the results of live-dead staining are shown in Figure 4A. Both groups were covered with a large number of living cells (showing green fluorescence) and a very few dead cells (showing red fluorescence), which proved that both groups had no obvious toxicity and good biocompatibility.

**FIGURE 5:**
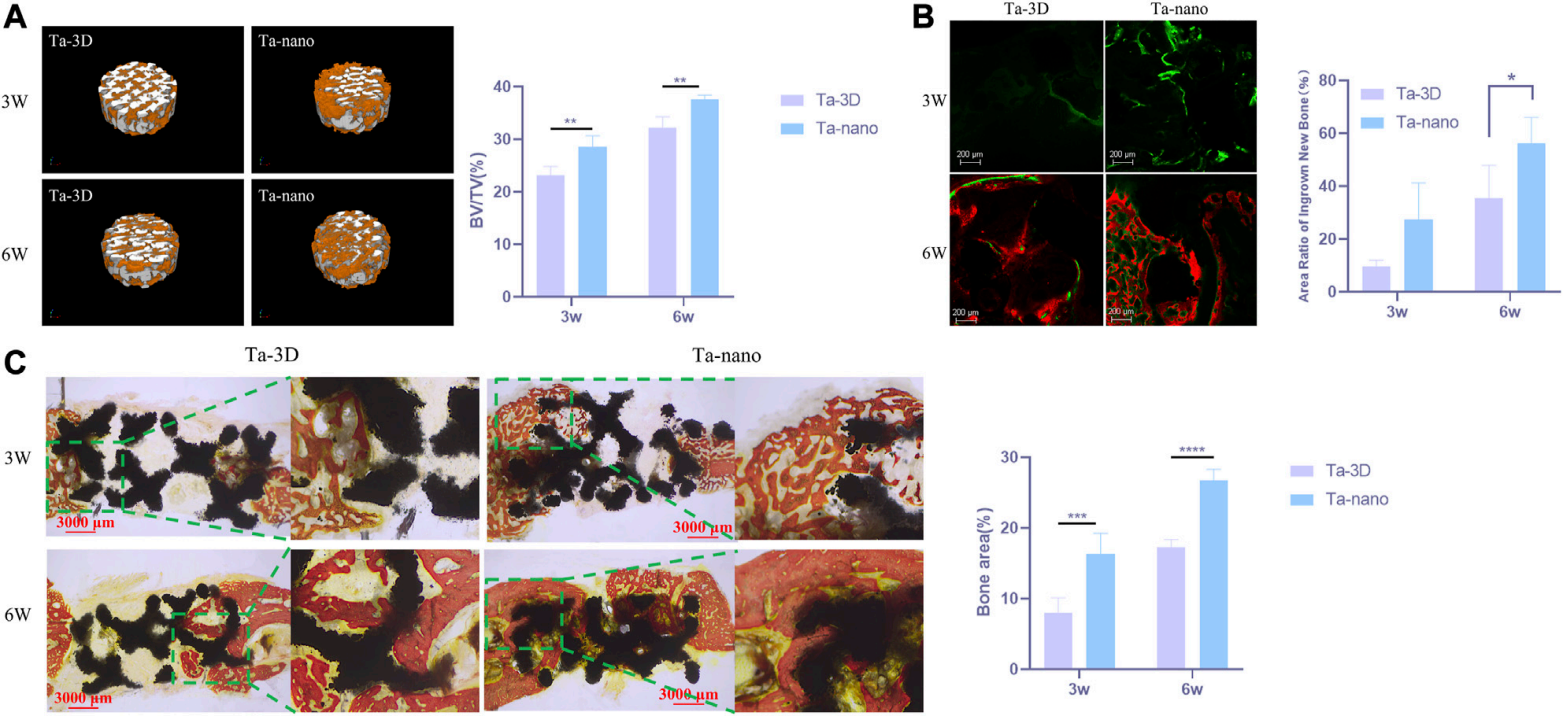
Capacity of New Bone Formation *In Vivo*. **(A)** Right: Micro-CT three-dimensional reconstruction of two groups of materials. Left: Percentage of the regenerated bone volume (BV) to the total volume (TV) according to the micro-CT images. **(B)** Fluorescent staining of porous scaffolds of new bone formation. The bone-formation fronts at the second, fourth week are marked by a green label from calcein green and red label from alizarin red respectively. **(C)** Histological section images of the Ta-3D and Ta-nano and percentage of osteogenic area of two groups of materials.P

After suffering from the bone defects resulting from pathological injuries, the self-repair of bone relies mainly on the amount of the cells rather than the volume. Therefore, a sufficient supply of cells (including MSCs and bone progenitor cells, etc.) is critical for effective bone regeneration (Li et al., 2022). CCK-8 was utilized to evaluate the adhesion and proliferation of BMSCs cells after 1/4/7 days of culturing on both groups of materials (Figure 4B). Figure 5 shows that there was no statistically significant difference between the two groups in day 1, but as the culturing time increased to 4 and 7 days, the proliferation activity of BMSCs on porous tantalum scaffolds in the Ta-nano group was significantly higher than that in the Ta-3D group, as the gap became more pronounced with increasing time, indicating that the nano-topographic surface structure enhance the proliferation of bone marrow mesenchymal stem cells indeed. This may be due to the fact that nanostructures are similar in size to Organelles and biomolecules, and cells can directly interact with them to form nanostructured matrices, which play a crucial role in stimulating cell proliferation (Babuska et al., 2022). Miyoshi and Adachi (2014) believes that nanostructures play a role as direct constraints of Integrin, which acts as a molecular link for transmission of a force between ECM and the actin filaments. The most characteristic way of Actin regulating cell proliferation is the tension stimulation of Actin Cytoskeleton involved in the Rho ROCK (Rho related protein kinase) pathway and the resulting Integrin aggregation pathway, which increases the activity of extracellular signal regulated kinase (ERK) and induces cell Cyclin D1 through ERK dependence, leading to enhanced cell growth (Assoian and Klein, 2008).

### 3.4 Osteogenic differentiation of BMSCs

**FIGURE 6:**
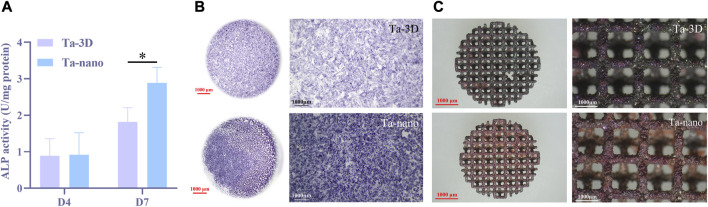
Osteogenic Differentiation of BMSCs. **(A)** ALP activity of BMSCs cells cultured on two groups of porous tantalum scaffolds on the 4 days and 7 days. **(B)** Representative staining ALP of BMSCs cells cultured on two groups of porous tantalum scaffolds on 7 days. **(C)** Representative staining ARS of BMSCs cells cultured on two groups of porous tantalum scaffolds on 21 days.

The ALP activity values of both groups increased with time, and the ALP osteogenic activity values on day 4 were slightly higher in the Ta-nano group than in the Ta-3D group, but there was no statistically significant difference between the two groups, while it could be seen that BMSCs incubated on the surface of the Ta-nano porous tantalum scaffold exhibited significantly better ALP activity than the Ta-3D group material without surface modification up to day 7, as shown in Figure 6A.

Figure 6B shows the representative images of ALP staining results on day 7 for two different groups of samples, respectively. Obviously, ALP staining level of the Ta-nano group is higher than that of the Ta-3D group without surface modification, which proves that the ALP osteogenic activity of the Ta-nano group is stronger than that of the porous tantalum scaffold without modification, which is consistent with the results of quantitative experiments.

ALP is an enzymatic protein secreted by osteoblasts whose active site consists of zinc-containing homodimeric metalloenzymes (Bremner and Beattie, 1995) which enables to hydrolyze a variety of phosphate compounds and release the inorganic phosphate (Pi) into the extracellular matrix (Farley et al., 1994). Besides, matrix vesicles also contain other calcium-dependent proteins which requires Pi to initiate crystalline nucleation of extracellular matrix calcium deposits, resulting in the formation of hydroxyapatite (Balcerzak et al., 2003). Consequently, the early expression of ALP of cells serves as one of the characteristic early markers of osteoblast differentiation, and the semi quantitative and staining results of ALP showed that nano surface modification contributed to the early osteogenic differentiation of BMSCs.

After culturing BMSCs cells on porous tantalum scaffolds of Ta-3D and Ta-nano groups for 21 days, the degree of mineralization of the extracellular matrix was further assessed by alizarin red staining. As can be seen from Figure 6C, red mineral compounds were observed on both groups of materials covering the internal lattices and surfaces of both scaffolds, and more mineralized nodules could be observed on the surface of porous tantalum scaffolds in the Ta-nano group. Alizarin red staining allows for the detection of calcium deposits and calcified nodules in cultured cells. The osseointegration of the implanted prosthesis with the surrounding bone *in vivo* consists of promoting the adsorption of biomolecules, recruiting BMSCs to adhere, differentiating into osteoblasts, and initiating the secretion of bone ECM, thus enhancing the early bone regeneration (Walmsley et al., 2015). Mineralization of the extracellular matrix is one of the processes of osteogenesis and implies late osteoblast differentiation. The BMSCs on the scaffolds of the Ta-nano group exhibited higher mineralization capacity, indicating that nano-topographic surface modification significantly enhanced osteogenic differentiation and mineralization of the cells.

### 3.5 Osteogenic gene/protein expression of BMSCs

**FIGURE 7:**
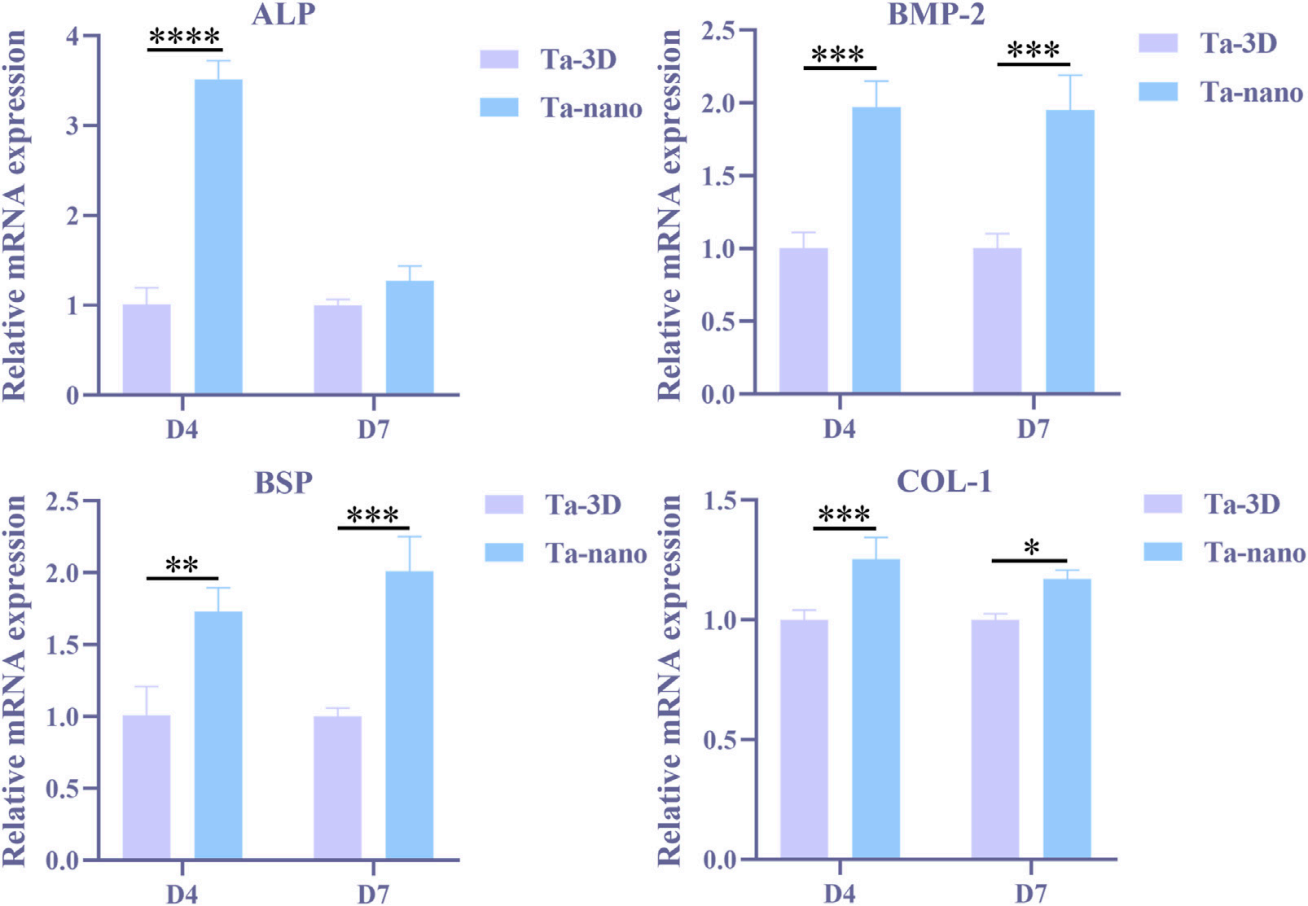
Osteogenic gene expressions (ALP, BMP-2, BSP and COL-1) of BMSCs on different samples at day 4/7 determined using RT-PCR.

Quantitative real-time quantitative PCR was performed to detect the expression of osteogenic genes, such as ALP, BSP, BMP-2 and COL-1 in BMSCs cultured on both groups of materials for 4 and 7 days, as shown in Figure 7. The expression of BMP-2, BSP and COL-1 was significantly increased in BMSCs cells of the Ta-nano group on days 4 and 7 compared to the Ta-3D group (*p* < 0.05). On the day 4, the amount of ALP gene expression was evidently higher in the Ta-nano group than in the Ta-3D group (*p* < 0.05), whereas no significant difference in ALP expression was observed between the Ta-3D and Ta-nano groups at day 7. Western blot analysis was used to detect the expression of COL-1 proteins in BMSCs cultured for 7 days, as shown in Supplementary Figure S1. The protein band grayscale of the Ta-nano group was deepened compared to the Ta-3D group, indicating a higher expression of COL-1, which was consistent with the gene expression result of RT-PCR. Compared with the differences in gene and protein expression between the two groups, it can be concluded that the nano-topographic surface can promote osteogenic differentiation of BMSCs.

The phenotype of osteoblasts is acquired in two stages. In the first stage, the cells proliferate and the matrix matures. Specific factors associated with the osteoblast phenotype, such as ALP, can be detected within 10 days of cell proliferation and matrix maturation or during 10–15 days. In the second phase of osteoblast phenotype acquisition, matrix mineralization, late osteogenic markers such as osteocalcin (OCN) are produced after 10–15 days or 25–30 days. Finally, multiple anabolic signaling pathways are actively involved in regulating bone formation, such as the BMP, Wnt and Runx2 pathways. RT-PCR results showed that ALP expression in nano-topographic surface modified porous tantalum material was higher than in the unprocessed group at day 4, while the difference in expression at day 7 was not significant. ALP is the most widely recognized biochemical marker representing osteoblast activity, and it is a typical protein for osteoblast phenotype and it is a typical protein product of osteoblast phenotype and osteoclast differentiation. In general, the appearance of ALP activity is an early phenotypic marker of osteoblastogenesis (Stein et al., 1990). The expression of BSP, COL-1 and BMP-2 in nano-topographic surface modified porous tantalum material was higher than that of the Ta-3D group in BMSCs cells cultured for 4 and 7 days. Bone sialoprotein (BSP), which appears after ALP expression, localizes to the mineralized matrix, and BSP helps nucleation of hydroxyapatite mineralization and promotes calcium admixture and formation of mineralized nodules (Gordon et al., 2007), which represents a phenotypic marker of osteoblast differentiation in the middle and late stages and is essential for initiation of bone mineralization and promotion of adhesion of osteoblasts to the mineralized matrix. Type I collagen (COL-1) is an extracellular matrix protein that stimulates osteoblast adhesion and differentiation (Mizuno and Kuboki, 2001) and is a mid-stage marker of osteogenic differentiation of bone marrow mesenchymal stem cells. Nano-topographic surface modification significantly promoted the type I collagen (COL-1) synthesis. Bone morphogenetic protein (BMP) plays an important role in bone formation and osteoblast differentiation by stimulating ALP activity and synthesis of proteoglycan, type I collagen, fibronectin and osteocalcin (An et al., 2016), and BMP-2 is an important growth factor that promotes the differentiation of BMSCs into osteoblasts and further boosts the osseointegration one of them. Therefore, nano-topographic surface modification serves to upregulate the expression of relevant osteogenic genes and enhance bone regeneration in both early and late stages of osteoblast differentiation and bone formation.

### 3.6 Capacity of new bone formation *In Vivo*

Micro-CT indicated that both groups of porous tantalum scaffolds at two time points were well integrated with the surrounding bone without infection or bone resorption, and the restorations did not dislodge and shift. The 3D reconstructed image of the cylindrical implant of the bone defect is shown in Figure 5A, which is approximately 2 mm high and 6 mm in diameter, from which it can be visualized that the amount of new bone around the restorations increased obviously with time. Afterwards, the volumes of the tissue area and bone tissue in this region were quantitatively analyzed for each material, and the bone volume fraction of each material showed that at both the third and sixth week, the amount of new bone production was significantly higher in the Ta-nano group than in the Ta-3D group, as shown in Figure 5A.

There was new bone formation around the material in both the Ta-3D and Ta-nano groups according to the fluorescence labeling results, where the width, brightness and fluorescence distribution area of the fluorescence strip around the restoration scaffold in the Ta-nano group were better than those in the Ta-3D group, which proved that there was more new bone formation (Figure 5B).

VG staining of histological sections at 3 and 6 weeks of restoration implantation (Figure 5C) showed that new bone production was already visible within the porous tantalum scaffold 3 weeks after the defect repair, while more bone production within the porous scaffold in the Ta-nano group. As the healing period was extended to 6 weeks, the amount of newly formed bone within the material increased significantly in both groups, and the nano-modified group remained superior to the unprocessed Ta-3D group. Histomorphometric analysis further confirmed the histological observations, showing a higher percentage of osteogenic area in the Ta-nano group at both time points.

Moreover, more new bone growth into the porous tantalum scaffold in the nano-topographic surface modified group was observed from the calcium xanthophyll-alizarin red double fluorescent labeling and Van Gieson staining experiments of rabbit mandibular defect implantation. Calcein and alizarin red were injected at 2/4 weeks postoperatively, and green and red fluorescence were observed in samples reared for 3 and 6 weeks, indicating that new bone production had already started at 2 weeks postoperatively.

In summary, the interface between cells and nanomaterials plays an important role in the physiological function of cells. Therefore, nano modification of the material surface to simulate natural bone structure and biological environment effectively enhances the osteogenic ability.

## 4 Conclusion

The 3D-printed porous tantalum scaffolds with microporous-like nano-topographic surface were prepared by alkali heat and water heat treatment. According to the cellular experiments, the nano-topographic surface modification by 3D printing enhances the adhesion and osteogenic differentiation of bone marrow mesenchymal stem cells. The *in vivo* experiments demonstrated that nano-topographic surface modification of 3D-printed prosthesis facilitates early and rapid osseointegration. A preliminary experimental validation was conducted for the application of nano-topographic surface modified porous tantalum prosthesis in the repair of bone defects in the jaws, which provides a theoretical and experimental basis for further clinical applications subsequently.

## Data Availability

The raw data supporting the conclusion of this article will be made available by the authors, without undue reservation.
