# Supplementary figures and images for: Osteogenic differentiation of 3D-printed porous tantalum with nano-topographic modification for repairing craniofacial bone defects
Source: Front Bioeng Biotechnol. 2023 Aug 21;11:1258030. doi: 10.3389/fbioe.2023.1258030 (PMC10475942; doi:10.3389/fbioe.2023.1258030)

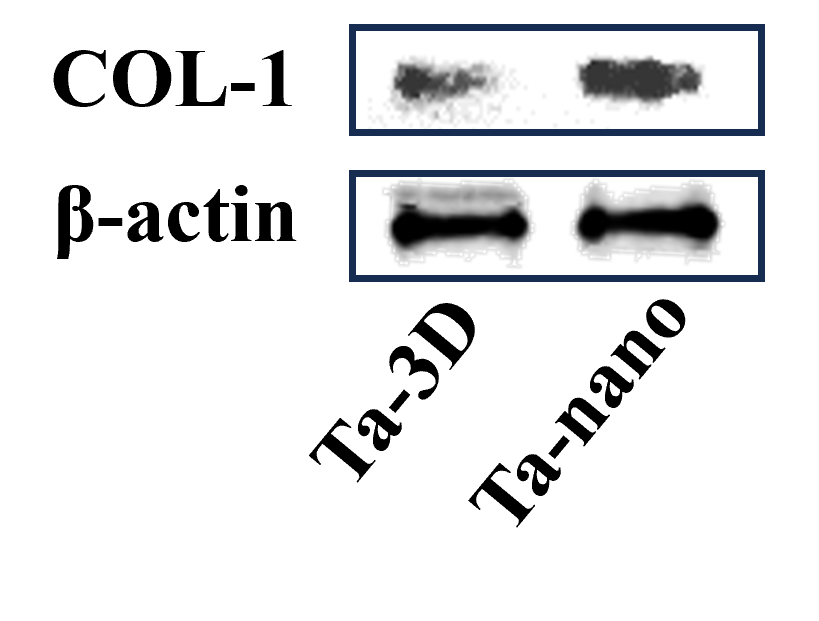

Supplement: Supplementary file 1 [file Image1.TIF]
